# The Prehospital assessment of severe trauma patients` performed by the specialist ambulance nurse in Sweden – a phenomenographic study

**DOI:** 10.1186/1757-7241-20-67

**Published:** 2012-09-18

**Authors:** Anna Abelsson, Lillemor Lindwall

**Affiliations:** 1 Department of Nursing, Karlstad University, Karlstad, Sweden; 2 Oslo and Akershus University College of Applied Sciences, Oslo, Norway

**Keywords:** Specialist ambulance nurse, Assessment, Prehospital care, Severe trauma, Phenomenography

## Abstract

**Background:**

A common feature of prehospital emergency care is the short and fragmentary patient encounters with increased demands for efficient and rapid treatment. Crucial decisions are often made and the premise is the specialist ambulance nurse’s ability to capture the situation instantaneously. The assessment is therefore a pre-requisite for decisions about appropriate actions. However, the low exposure to severe trauma cases in Sweden leads to vulnerability for the specialist ambulance nurse, which makes the assessment more difficult. Our objective was to describe specialist ambulance nurses’ perceptions of assessing patients exposed to severe trauma.

**Methods:**

This study had a phenomenographic approach and was performed in 2011 as an interview study. 15 specialist ambulance nurses with a minimum of 2.5 years of experience from praxis were included. The analysis of data was performed using phenomenography according to Marton.

**Results:**

The perceptions of assessing patients exposed to severe trauma were divided into: To be prepared for emergency situations, Confidence in one’s own leadership and Developing professional knowledge.

**Conclusions:**

This study reveals that the specialist ambulance nurse, on the scene of accident, finds the task of assessment of severe trauma patients difficult and complicated. In some cases, even exceeding what they feel competent to accomplish. The specialist ambulance nurses feel that no trauma scenarios are alike and that more practical skills, more training, exercise and feedback are needed.

## Background

A common feature of prehospital emergency care is the short and fragmentary patient encounters with increased demands for efficient and rapid treatment [1]. Crucial decisions are often made and the premise is the specialist ambulance nurse’s (SAN) ability to capture the situation instantly [2]. The assessment is therefore a pre-requisite for making decisions about appropriate actions [3]. The assessment results in well-planned emergency care of the patient in order to stabilize vital signs and maintain vital functions [4]. In countries with high incidence of severe trauma cases, the ambulance personnel maintain and increase their skills when handling severe trauma. The same effect cannot be achieved in Sweden because of the infrequency of severe trauma cases. Presently, severe trauma patients represent only 2–3% of patients receiving emergency ambulance care in Sweden. The low exposure to severe trauma cases could lead to vulnerability, which makes the assessment more difficult [3,5]. In Sweden the SAN has the highest degree of training and is thereby the medically responsible professional at the prehospital trauma site. The SAN may, therefore, find themselves in what can be describes as "caring solitude" [3] or "the small caring team's vulnerability" [6]. This means that there are natural limitations to assessment and care-giving, so these can differ as a consequence of the team consisting of only two people; a SAN and a basic emergency medical technician (B-EMT) or a registered nurse (RN).

The education of the Swedish ambulance workforce consists of three levels. The B-EMT has a high school diploma or equivalent and do not administer drugs. The RN has a 3 year bachelor degree and is qualified to administer drugs. The SAN is a registered nurse with the same 3 year university degree, in addition to hospital experience. Furthermore, the SAN has an additional one year specialist training at a university with focus on prehospital care. The SAN is a protected professional title [7] and in many cases also includes a master's degree in prehospital emergency care. All three levels are in most cases trained in prehospital trauma life support (PHTLS). From 2005, all ambulances in Sweden are staffed by at least one RN or SAN who can administer drugs [8]. There are no national requirements for personnel in the ambulance service to undergo any formal preparation, have any continuing professional development or revalidate their knowledge and skills. In metropolitan areas there is access to emergency physicians who may provide pre-hospital support.

However, since Sweden has a population density of 23 inhabitants per square kilometer (60 per square miles) [9] the majority of the population will be assessed by the ambulance personnel with SAN as the highest medically responsible person on the scene of accident.

The emergency ambulance care in Sweden is a relatively unexplored area [10]. A literature review shows that there are few known published studies that illustrate the knowledge of the SAN. The cause of the limited research in this area in Sweden may be the fact that the registered nurse did not enter the scene of prehospital care until the past few decades. Experience of severe trauma assessments is an unexplored area at the present time. As a profession, the SAN is a relatively new specialty and the number of SANs being employed has increased, which leads to an interest in research in this field. *The purpose* of this study was to describe specialist ambulance nurses’ perceptions of assessing patients exposed to severe trauma.

## Methods

Based on the purpose of this study, a phenomenographic design according to Marton [11] was chosen. Phenomenography is based on capturing and describing thoughts about the experience of phenomena [12]. The phenomena in this study are assessing patients exposed to severe trauma. The phenomena in the lived world often represent an unconscious thought that has not previously been expressed or reflected on [11,12] and the researcher hopes that the participants will reflect on their experience of the phenomena [11]. Phenomenography highlights the collective perception of a particular phenomenon. The intention is to describe variations of perceptions from the perspective of a variety of experiences [13]. In phenomenography, the researcher works with first- and second-order perspectives. The first-order perspective – the structural – represents what something is, i.e. the facts [12,14]. Second-order perspective – the referential – means how the phenomenon is perceived [11,13].

### Participants

The participants consisted of 15 specialist ambulance nurses recruited from varying parts of two counties in Sweden. In accordance with phenomenography, the participants were strategically selected to obtain a variation in the size of the district covered [12,13,15] (see Table 1). The participants were asked to take part in the study by the chief of ambulance, who explained the purpose of the study verbally and provided each potential participant with written information. The contact details were then forwarded to the first author who contacted the participant. The inclusion criteria were specialist ambulance nurses who had a minimum of 2.5 years of experience working as such. The assessments done in the group varied by a mean of 6 times per year.

**Table 1:** Demographic and educational characteristics of the participants

	
Sex	Male/female 7/8
Age	Between 32–53 years, mean age 39 years
Years worked as an RN before becoming an SAN	Between 5–25 years, mean 9.5 years
Years worked as an SAN	Between 2.5-12 years, mean 4.5 years

### Data collection

The participants who accepted to be interviewed were contacted and a time for interview was agreed upon. The locations for the interviews were at the participants’ fire or ambulance station. During the interview, the participants were informed about the voluntary nature and that they could withdraw at any time. Furthermore, the interview data would be treated confidentially and it would not be possible to identify the participants. Before inclusion, written consent was obtained from all participants. The participants were not dependent on the researchers in any way.

Within phenomenography, the most common data collection form is an interview [11]. A qualitative, explorative design with phenomenographic approach was selected to explore the variations in the specialist ambulance nurses’ perceptions of assessment of patients exposed to severe trauma (see Table 2). The first author conducted the interviews over a 5-month period. A pilot interview was performed to test the validity of the questions. Both authors assessed the questions and minor changes were made. The pilot interview was included in the data. In this study, we used opening questions as: ‘How often do you assess patients exposed to trauma?’ and ‘Would you like to tell me about any situation in which you cared for a patient exposed to severe trauma?’ These were followed by probing questions such as: ‘Can you give an example?’ and ‘Can you describe what you mean?’ The interviews were audio recorded and transcribed verbatim. The duration of the interviews were between 40 to 70 minutes and captured how the specialist ambulance nurses perceive the assessment of patients exposed to severe trauma. Quotes from the text are presented in such a way that informants cannot be identified. Only the two authors have read the interview material [16].

**Table 2:** Data analysis in seven steps according to the phenomenographic tradition modified by the authors

	
1.	Familiarization. Interviews were read through to become familiar with the material.
2.	Condensation. Statements that shed light on the researched phenomenon were highlighted.
3.	Comparison. Statements were systematically compared with each other to identify similarities and differences.
4.	Grouping. Data that appeared to be similar were grouped.
5.	Articulating. The essence of similarity within each group was described. Groups were then put together based on the internal relations of the statements whereby referential themes were formed.
6.	Labeling. Themes were clarified by formulating suitable vocabulary, and structural themes were established.
7.	Contrasting. Comparison, focusing on similarities in order to ensure that each theme had a unique character.

Throughout the analysis process, a reiterating process was used, especially between steps four and five. Quotes were chosen to strengthen the description of the referential themes [15].

### Analysis

#### Ethical approval

Approval for the study was obtained through the Local University Research Ethics Committee. Permission was obtained from the head managers in the counties. Informed consent was obtained from all participants, who were also informed that they could withdraw from the study at any time.

## Results

The result describes a variety of perceptions of the phenomena to assess patients exposed to severe trauma. The structural themes were; To be prepared for emergency situations, Confidence in one’s own leadership and Developing professional knowledge, (see Table 3).

**Table 3:** Descriptive structural and referential theme related to assessing patients exposed to severe trauma

**Structural themes**	**Referential themes**
**To be prepared for emergency situations**	Focus on overview of accident site and patient and on need of feedback
	*To observe the situation* -*Ready to act Confirmation* and *Validation of one’s knowledge*
**Confidence in one’s own leadership**	Focus on handling leadership.
	*Responsibility of leadership in emergency situations* and
	*Combining theory and practice*
**Developing professional knowledge**	Focus on how the work strategy changes based on the competency of the colleague and the need for competence and skills development.
	*Lack of a prehospital work strategy, Supporting novices, - Being alone,Being familiar with the equipment, Seeking new knowledge* and *Practicing critical scenarios*

To be prepared for emergency situations focused on the ability to assess emergency situations and on evaluating and inquiring for confirmation about prior experience. The referential themes were: *To observe the situation, Ready to act, Confirmation* and *Validation of one’s knowledge.*

*To observe the situation* meant to, on arrival at the scene of accident, create an image of the surroundings and of the influencing factors of the trauma. It was considered important to find out what had happened at the time of injury in order to interpret the amount of violence the body had been exposed to and how the energy of the impact had injured the patient. The body’s position and appearance in relation to a vehicle’s appearance or objects that may have injured the body was considered in the assessments. The ability to read the scene of the accident and the violence the patient suffered was based on theoretical knowledge from the specialist education and from experience of having seen accidents and patients exposed to severe trauma before. The experience resulted in acquired knowledge about injuries that could be expected from reviewing the scene of the accident. One SAN said: *You need to see the big picture and see the entire accident … When you see the patient for the first time, you know a lot already*. Another said: … *you can figure out that he may have penetrated every possible organ, and he has internal and external bleeding, so it is as much an emergency as it can get*.

*Ready to act* meant having worked with severely ill patients in the hospital, which had resulted in the acquired experience that the prehospital assessment was based on. Working at a hospital had provided the opportunity to review and reflect on the differential diagnosis in a safe environment, which was described as necessary. A single SAN often performed prehospital assessment. As the assessments rarely occurred, the SAN did not obtain proficiency. This meant that the SAN was ready to react, but did not feel safe in the situation. Theoretical knowledge from the specialist education, along with practical experience that solidified the theory, provided a sense of flexibility in the assessment. The overview of the patient and the situation was described as a feeling of knowing whether a patient was in critical condition or not. The reflective mindset that evolved provided an opportunity to take a step back and reassess patients who were not responding to the treatment as expected. The experience resulted in humility and confidence in the fact that not everything always goes as expected. It is possible to be wrong and have the courage to say it out loud. The experience also led to an avoidance of tunnel vision and not being paralyzed, which opens up for more information being processed simultaneously. One SAN said: *You suspect what the problem might be. What is it that I am looking at? What is the cause? … Sometimes when things are very critical you just handle the symptoms and treat the symptoms … You don’t have time to think. You still have a … I have worked for such a long time … more than 25 years, I have learned a thing or two during these years. I have seen a lot, but I still try to think and not get caught in the same pattern of thought, which otherwise happens easily.* Another SAN said: *In situations like this, nothing is perfect and everyone is doing their best. It's very important to remember that you can only improve and things will never be perfect. You can never encounter the same situation again. You can never practice enough in a situation, because this particular situation may never happen again. You simply have to do your best*.

In *Confirmation* it was revealed that there is a need for confirmation for whether the assessment was right or wrong. Partly in order to process the accident and get closure, but the feedback also provided knowledge and experience for the next event. The thorough knowledge and experience of SANs provided the opportunity for reflection on how patient care was conducted. Discussions with an experienced SAN were used as a control mechanism and confirmation of the approach used. By evaluating whether one’s own actions were consistent with those of others, a sense of confirmation was reached. The subsequent talks with emergency room staff aimed at knowing the patient’s injuries. The information helped improve the treatment of patients with similar injuries. If the feedback and self-evaluation of the assessment was not done, there was always a risk of continually assessing patients wrongly. One SAN said: *But I think it's a very serious dilemma … Serious that I never get any feedback. I may have gone for 4 years and treated patients completely wrong … I want to know whether I am right, think right and whether I'm on the right track.* Another said: *… it's about skills and experiences. I sit with a competent colleague and she says, Yes, you have done this and that and you know what has happened inside the body. He had not survived and you were thinking right … then I feel safe. Why and how, and these thoughts, if you have a colleague, they would tell you that you are right and that's what you want to hear.*

*Validation of one’s knowledge* focused on the SANs knowing their own skills and working according to treatment guidelines. The guidelines were supportive of the assessment and facilitated the management of responsibility. These were sometimes experienced as restrictive or not sufficiently clear, because the prehospital environment differs beyond what could be predicted. Rapid decisions taken at the incident site were not always supported by guidelines, which subsequently could be challenged by the receiving hospital. This was a result of vaguely implemented boundaries of tasks. One SAN said: *Inside the hospital, I know exactly what I can and cannot do. Here I do not know. I should know more what I am allowed to do and what not … Right now, it's very unclear. What is the emergency medical service and what is the specialist ambulance nurse allowed to do? What kind of assessments can you do and what do you say yes or no to? There is no such thing, I would like to have it written down on a piece of paper. These decisions may be made or not.*

Confidence in one’s own leadership focused on the position of leadership that the SAN title entailed. The referential themes were: *Responsibility of leadership in emergency situations* and *Combining theory and practice.*

The *Responsibility of leadership in emergency situations* was described as how the SAN as the highest prehospital medical profession holds a leadership role at the scene of accident. With the safety of personnel and patient care as the priority, the SAN should with authority, be able to direct the fire brigade, police and additional ambulances in how the patient related work at the site should be a prioritized. This was described as a big responsibility. Improved quality of work was achieved when they were given a few minutes of preparation for what awaits at the scene of accident, as opposed to arriving at the site with neither time nor opportunity to prepare mentally. Having the fire brigade at their disposal at the scene of accident was described as providing a sense of security. Working with competent staff made the delegation quick and smooth. It was also described as stressful when the SAN wanted to focus on the patient while the fire brigade at the same time wanted to know what to do next or when they wanted to work faster than SAN saw fit. Experience from working at the scene of accident helped when handling the stress and resulted in daring to take the time to examine the patient. Keeping a distance from the scene also allowed them to retain a complete picture. The experience of having handled the situation at the scene of accident offered a retrospective sense of poise, which made the SANs grow in their role. One SAN said: *It's hard when everybody requires your attention. … It's been very hard when I feel like I have 10 fire fighters and four ambulances that look at me and I need to make a decision. I think it's terribly hard and I've been through it a few times and then it's just that I feel that the whole world is on my shoulders and right there and then it actually is! That actually makes it very difficult and I think that's really demanding.*

In *Combining theory and practice* the specialist education was considered to be a pre-requisite for accomplishing a proper assessment. The level of education of the staff was described as having to match the requirements set for the prehospital treatment, regardless of the amount of time for the transport. The theoretical knowledge that the specialist education provided solidified the prehospital work. The education needed to rest on the experience of the SAN as a previous nurse to provide a broader analytical approach and create a new thought pattern. The theory provided by the education created a clinical eye that led to a faster and more accurate assessment effort. Discussions during the education provided experiences that guided the SAN’s assessment and treatment. Subsequent exercises of assessment and treatment of trauma resulted in the theory being absorbed and transforming to experience. The knowledge could be structured, which in turn provided a more secure assessment. One SAN said: *There are really good people coming in, but they do not have the practical skills for what and how to do things. If you want to work in an ambulance then perhaps you should have an education, this should be a requirement … You can’t work at an ICU today if you don’t have a specialist education in intensive care. But in the ambulance, you may have worked as a registered nurse in Community Care… We are first and we are alone.* Another SAN said*: The thing that has made me stronger, that's the whole specialist education. It carried me into a higher league … There is a clear relevance to reality. You elevate the theory and summarize it in the exercises, to see how the reality is. You cannot do this with all types of knowledge, but I'm very grateful for such an education.*

Developing professional knowledge focuses on the uncertainty in cooperation with other professions and learning to be able to meet new challenges.

The referential themes were: *Lack of a prehospital work strategy, Supporting novices, Being alone, Being familiar with the equipment, Seeking new knowledge* and *Practicing critical scenarios.*

*Lack of a prehospital work strategy* describes strength in working with personnel from other specialties, such as intensive care and nurse anesthetist, when it came to discussions and reflections on physiology and medicine. However, there was an absence of prehospital work and practical experience in the ambulance service. Even the doctors were considered to provide hospital-adapted care and therefore came short in the prehospital settings. A SAN said: *She had to let it go (patient responsibility). She did not understand. She wanted to get the others out of the car as well. You just had to say, we will not get them out, it’s too late. We must focus everything we have on this one if we are to have any survivors. She accepted it. There was nothing more to it… It went well, she did what I asked her to. She knew that she didn’t have much to offer because she has never met such a patient in the field before. She has seen them on the operating table, but not … where they are not stable. Where you do not have access ports on the patient. You basically don’t have anything.*

*Supporting novices* was described as how the SAN took charge at the scene of accident when inexperienced RNs were subjected to many impressions and having a subordinate role was enough. Inexperienced RNs had no prehospital skills to oversee and plan the work, but instead wanted to load up and leave without having assessed and treated the patient. A need to educate, support and mentor the new RN was expressed, in order to create a safer working environment for them. One SAN said: *Some are new and you have to provide support … if you feel you cannot handle it, you can hand it over to a more experienced colleague.*

*Being alone* was described as a shortcoming when much should be done at the incident site and the SAN feels inadequate. A sense of security emerged in the work with colleagues with the same specialist education. It eased the feeling of being alone and vulnerable. Two SANs could complement each other and work side by side, based on having similar thinking patterns and the same long-term planning of the procedures. Ideas and thoughts were reflected upon at an advanced level to check and confirm the choice of strategies. Care-giving went smoother because tasks did not have to be delegated or validated when both had the same knowledge and medical responsibility. The feeling of being equal was described as opposed to having a none registered nurse as assistant. It also reduced the risk of tasks being forgotten or getting stuck in a single pattern of thought. One SAN said: *It does happen that it is a life or death situation. Then you may feel that … this is up to me. If I have a specialist ambulance nurse with me, then there are two of us and it provides a sense of security … you work better together as well … Then a jumbo jet could crash … It doesn’t matter what kind of emergencies we get, we will handle them one way or another, to the best of our abilities.* Another SAN said: … *I usually think of when working with him (SAN) that we are doing the same things, but in different places, and we are in full control … When I work with EMT, they do not know what is wrong with the patient and this makes me feel very alone. That is if other ambulances don’t get there as well … You don’t have any decision support. … I make a decision … but I have no one to consult with. I can ask but I get no response … that is how it feels, imagine if everyone had the same training.*

*Being familiar with the equipment* in the ambulance was considered important. Being comfortable with the use of the equipment created a sense of security that resulted in full utilization of the equipment. The knowledge had come through the exercises in the specialist education and in practical work with more experienced staff. The continuous practice and use of the equipment sharpened and maintained the skills with the equipment. One SAN reported: *Some equipment is used seldom, and once there is an emergency you feel uncertain. It's usually someone who knows the equipment, but it would feel safer if you had practiced more.*

*Seeking new knowledge* is described as a need for constant education. Reading scientific literature and articles could maintain and update the knowledge. As each patient and situation was perceived as unique, a gap in knowledge was often encountered. Following the current developments prevented the feeling of uncertainty as well as satisfying the need for being well informed and competent. One SAN said: *You will never know everything. You are always learning. You can work for 20 and 40 years and still one day you will come across a patient case whom you have never before encountered. Maybe that is why you can never stop searching for knowledge. It's hard to say exactly what you should become better at, but … you should not stop searching for knowledge. You have to continue, like sucking up all the knowledge you can.*

*Practicing critical scenarios* focuses on difficulties in making assessment into a routine task in cases of severe trauma. It is described as not regularly practiced and each situation was experienced as unique. A sense of uncertainty was raised about not being able to handle the situation, but still being expected to know everything. Exercises were described as an option to increase competency. Various patient cases followed by group discussions could improve, correct and refine the assessments. The exercises should be aimed at better and safer care of patients. One SAN said: *For the patient's sake, we need all sorts of exercises on all possible levels. But I have solved the situations I have been faced with. One can certainly provide a better solution but we have still solved them.* Another SAN said*: I am interested in practicing, you feel safer and then you can rest assured in that, I know I can do this and then you may improvise …*

## Discussion

The results arise an awareness of how difficult the severe trauma scenarios are perceived for the specialist ambulance nurses who have to perform assessments and treatments in very complicated clinical cases of severe trauma without the availability of doctors and other specialists in the hospital. A country with such a scattered population as Sweden, therefore, needs to complement their pre-hospital care with competent and experienced professionals as the SAN.

There are few studies with focus on assessment conducted by the SAN [2,5,17]. The result of this study shows SAN's perception of assessing patients exposed to severe trauma, which have not previously been studied. Studies have been carried out on ambulance personnel [1,18,19] and on the patient's experience [20] of the assessment situation, which our study complements with an additional dimension, SAN's experience and how this affects the assessment.

Perceptions of knowledge gaps and the fact that no trauma scenarios are alike suggests a lack of experience regarding the assessment conducted relatively infrequently, on a mean of 6 times per year for the participants in the study. It is described as difficult to create and maintain experience and there was a sense of insecurity in the assessment situation. Ignorance of what awaited on the site of the accident, however, created a sense of preparedness of SAN to handle the situation. This is in addition to the research that has shown that the ability to make decisions and act autonomously are properties of the SAN that enables quick assessment of patients with life-threatening conditions [7,21]. A feeling of being prepared for future trauma cases is supported by Elmqvist [1] and resulted in a humility and understanding that the emergency care does not always end as expected. But it created a need for confirming one’s actions. Discussions with other SANs opened for reflections on an advanced level. Research shows that discussions should take place with others who have been in similar situations and therefore understands the difficulties in pre-hospital care [1,6]. The discussions served as a verification of that the assessments were correct and optimal actions were performed. New knowledge and experience was created by reflecting on the events, which are corroborated by previous research [22,23] as well as included in the SAN’s competence description [7]. From this, arises an awareness that further research is needed on how structured discussions between colleagues may enhance the practical acquiring of knowledge and experience in the profession. The desire for confirmation also reveals a need for more support from higher medical qualification. To routinely provide feedback from prehospital trained doctors for severe trauma cases could enhance the performance of SAN. Today, this area is unexplored and need to be studied in order for the feedback to develop, refine, and verify SAN`s assessment skills with increased patient safety as a result.

Treatment guidelines are perceived as restrictive and unclear due to the prehospital environment being unpredictable. Fast decisions are based on very little information, which is also stated by Wireklint [19]. In some cases the assessment resulted in actions that were not supported by the treatment guidelines. The ambulance service often has access to consultation by telephone with a hospital-based physician, however, in severe trauma cases there is rarely time. The SAN as a profession therefore, needs to be adapted for the increased challenges and higher demand for independence [7], which could decrease limitations in patient care.

SAN shall, together with the fire brigade and police create a safe environment at the scene of accident [7]. Working with the fire brigade gave SAN a sense of security, but could also be experienced as stressful. Previous experiences of responsibility for leadership at the scene of accident determined how SAN managed the balance between fire brigade’s desire to work faster and their own need to take the time to assess the patient. If there are well-defined boundaries between ambulance, police and fire brigade, a sense of trust is created in each other’s capabilities and roles. If the different groups on the ground have insight into each other's knowledge, understanding and respect is created between professions. Only then, the team can together contribute to a secure environment for the patient [1,24]. A feeling of increased self-confidence is described as a result of having mastered this situation. Together with exercise, this could increase the number of SANs with knowledge and courage to handle the responsibility at the scene of accident. These findings call for future research on how the SAN can be trained in practicalities of leadership at the site of accident.

The prehospital work is perceived to be based on knowledge, experience and a prehospital specialist education. Theoretical knowledge from the specialist education was reflected in the prehospital environment. To have practiced the assessment and treatment of patients exposed to severe trauma, during the specialist training, transformed theory into practical experience. Research on exercises and training conducted by SAN is limited. Research needs to be done on how the exercise should be executed in the organization to maintain skills and create more experience in the assessment and care of the patient exposed to severe trauma.

Being two SANs mitigated the feeling of loneliness. To discuss and reflect on different tasks with each other was perceived as complementing and beneficial. This is consistent with previous studies that describe the loneliness of the ambulance crew and how they therefore, rely on and have high expectations of each other's knowledge [3,6]. The assessment is reinforced by an interaction between people who complement each other [19]. Being well educated and continually seeking new knowledge, which is also part of the SAN’s profession [7] provided a sense of security. However, Siriwardena [25] states that prehospital personnel need to use research more frequently. Participating in research will strengthen evidence-based emergency medical services and thus provide the best care for the patients.

### Methodological considerations

As a limitation, we need to highlight the differences in the prehospital care setting in Sweden compared to those in other countries. SAN is specially trained in prehospital care working independently and is responsible for assessment and treatment of patients.

Phenomenography is a research method and in this case, we chose it because of its way of capturing the participants’ spontaneous thoughts about the assessment of patients exposed to severe trauma and thereby acquiring knowledge about their way of experiencing the world. A difficulty in health care is that people’s experience of the world varies. Phenomenography can provide the discipline of nursing with knowledge about variations in how nurses think and how phenomena are experienced in nursing. The availability of descriptions of nurses’ perceptions of core professional settings can be considered as a resource for enhanced awareness of the nursing profession [12].

The credibility in this phenomenographic study is based on the relationship between the data and the categories. The differences and similarities are well supported by the material, which clearly shows in the results [12]. The relevance of the categories is supported by excerpts from the interviews [12,13]. The validity is emphasized by the fact that the study has investigated the intended purposed and that the results reflect the phenomena. Thereby, the goal of the study has been fulfilled [14]. One author, with expertise in the field, conducted the interviews. The validity of the material increases when the author has experience in the profession as a specialist ambulance nurse. This pre-understanding of the profession can promote the vision and help to recognize and find patterns and facts, veiled as they may be [14]. On the contrary, one can argue that the authors’ understanding of the phenomena may be a weakness. This in turn will cause the author to only see the expected. The first author therefore chose to read the interviews and carry out an initial analysis and then analyze and process the material together with the second author. The fact that the author conducted the analysis in dialogue with another researcher provides a more complete outcome space to the analysis, which strengthens the reliability. In order to increase the reliability even further, the results were verified with an additional SAN [12,14].

## Conclusion

This study reveals that the SAN, on the scene of accident, finds the task of assessment of severe trauma patients difficult and complicated. In some cases, exceeding what they feel competent to accomplish. The SAN feel that no trauma scenarios are alike and that more practical skills, more training, exercise and feed-back is needed.

## Competing interests

The authors declare that they have no competing interests.

## Authors’ contributions

The first author conducted the interviews, analyzed the material and wrote the manuscript. The second author supervised in making interview questions appropriate and was the additional evaluator in the categorization procedure and in the writing of the manuscript. All authors read and approved the final manuscript.
